# Sprint and jump performance in elite male soccer players following a 10-week Nordic Hamstring exercise Protocol: a randomised pilot study

**DOI:** 10.1186/s13104-017-2986-x

**Published:** 2017-12-04

**Authors:** K. Krommes, J. Petersen, M. B. Nielsen, P. Aagaard, P. Hölmich, K. Thorborg

**Affiliations:** 1 Sports Orthopedic Research Center-Copenhagen (SORC-C), Department of Orthopedic Surgery, Copenhagen University Hospital, Kettegaard Alle 30, Hvidovre, Denmark; 2 Physical Medicine & Rehabilitation Research-Copenhagen (PMR-C), Department of Physical and Occupational Therapy, University of Copenhagen, Copenhagen, Denmark; 3 Department of Radiology, Section of Ultrasound, Diagnostic Centre, Rigshospitalet, Faculty of Health Sciences, University of Copenhagen, Copenhagen, Denmark; 4 Department of Sports Science and Clinical Biomechanics, University of Southern Denmark, Odense, Denmark

**Keywords:** Nordic Hamstring exercise, Hamstring strain injuries, Soccer, Football, Eccentric

## Abstract

**Objective:**

The preseason Nordic Hamstring Protocol (NHP) reduces hamstring strain injuries in football players. Despite persisting injury rates, elite clubs are reluctant to apply the NHP often over concerns of negative impacts on performance. This pilot study investigated if sprint or jump-performance outcomes tended to increase or decrease following implementation of the NHP in elite male soccer-players.

**Results:**

Nineteen male soccer players from the Danish 1st division were randomised to perform NHP (27 sessions) during pre-season, or to control group (CG). Sprint performance (30 m with 5 and 10 m split times) and countermovement jump (CMJ height) was measured before the mid-seasonal break and again after 10 weeks of performing the NHP at the end of pre-season. Dropouts were due to transfers and injuries unrelated to performing NHP (NHP = 0, CG = 5). Sprint performance on the short split distances improved for most players in the NHP (6 out of 9 improved, median changes for 5 m split: − 0.068 s; 10 m split: − 0.078 s), but not CG (2 out of 5 improved, median changes for 5 m split: + 0.1 s; 10 m split: CG: + 0.11 s), but both groups had small declines at 30 m sprint (NHP: 7 out of 9 declined, median changes: + 0.116 s; CG: 4 out of 5 declined, median changes: + 0.159 s). CMJ height mostly improved in both groups (NHP: 6 out of 9 improved, median changes: + 2.1 cm; CG: 4 out of 8 improved, median changes: + 0.55 cm). Performing the NHP in elite soccer players did therefore not seem to negatively affect sprint and vertical jump performance outcomes in the present study, while in fact showing some promise for the more explosive characteristics such as the short 5 and 10 m split-times and maximal CMJ height, which all are highly relevant performance parameters in elite football.

## Introduction

Hamstring injuries are common in sports involving sprinting and jumping, including different variations of football [1–4]. Preventing new and recurrent hamstring injuries in amateur and sub-elite football has effectively been achieved in several trials by implementation of the Nordic Hamstring Protocol, a 10-week pre-season eccentric hamstring strength-training protocol based on the Nordic Hamstring exercise [5, 6]. Elite clubs are familiar with the exercise but only few utilize the full protocol [7], more than half have reservations about the exercise [7], and a large proportion do not employ it in any way [8]. This is in line with epidemiological data showing maintained or even slightly increased incidence of hamstring injuries in elite clubs over the past decade [9]. It is suggested that elite football environments holds certain specific barriers to implementing preventive measures [10–12], and some have even argued that the Nordic Hamstring exercise can decrease performance and prompt injuries [13, 14]. Others propose that instead of the Nordic Hamstring exercise, other exercises should be employed in sprint-training and hamstring injury prevention, although no data supports this claim [15–17].

The Nordic Hamstring Protocol in isolation increases eccentric hamstring strength, which is considered essential for sprint performance [18], and studies using other means to increase eccentric hamstring strength have indeed also reported improvements in jump [19] and sprint [19, 20] performance. The Nordic Hamstring Protocol has not been investigated for its isolated effect on jump or sprint outcomes but studies employing the exercise in either various dosages or different timings, or accompanying other forms of training, have indicated either maintained or increased jump or sprint performance, along with gains in eccentric hamstring strength [21–28]. The main purpose of this study was to pilot implementation of the Nordic Hamstring Protocol on team-level, in order to monitor its effect on sprinting and jumping performance in elite male football players. The secondary purpose was to obtain data for sample size calculations, and other useful information for future research.

## Main text

### Methods

Potential participants were 25 football players in a first team squad from the Danish 1st Division, chosen based on convenience sampling as one author (JP) served as Team Doctor for the team. Injured players at the time of pre-testing were excluded. The season in the Danish 1st Division starts in August and ends in June, including a mid-seasonal break from December to March. All pre-tests were performed in the week following the final match in November 2008. The 10-week intervention was introduced when physical training started in January. Post-tests were performed in the week prior to the first played match in March 2009. Besides the intervention, both groups followed usual diet- and exercise regimes. One author stratified all players according to age and playing position, and subsequently randomised them to intervention (Nordic group) or control group, by drawing lots in blocks of two matched players from an opaque envelope. The same author carried out physical tests, and sprint and jump tests, which were all completed on separate days. The reporting of this study follows the Consolidated Standards of Reporting Trials (CONSORT) statement when applicable [29].

#### Intervention; the Nordic Hamstring Protocol

The Nordic Hamstring Protocol consists of 27 sessions of the Nordic Hamstring exercise, performed before regular warm-up during a 10-week period, starting with 1 weekly session of 2 sets of 5 repetitions, and ending with 3 weekly sessions with 3 sets of 12, 10 and 8 repetitions respectively in week 5 through 10 [30]. If players were not attending a training session, they were instructed to perform the protocol at home. The Nordic Hamstring exercise is a partner exercise where the player attempts to resist a forward-falling motion using his hamstrings to maximize loading in the eccentric phase. The player were asked to keep their hips fixed in a slightly flexed position throughout the whole range of motion, and to brake the forward fall for as long as possible using their hamstrings, and to try keeping tension in their hamstrings even after they have to ‘‘let go’’. They were asked to use their arms and hands to buffer the fall, let the chest touch the surface, and immediately get back to the starting position by forcefully pushing with their hands to minimize loading in the concentric phase. The Nordic Hamstring Protocol has been described in detail by Mjølsnes et al. [30].

#### Sprint testing

Sprint performance was assessed on an indoor all-weather-track to ensure similar ground and weather conditions between sessions. A warm-up program similar to the program used before match-play preceded the running tests, which consisted of 20 min of various running drills without ball led by the captain of the squad. Photocells were positioned at 5, 10 and 30 m for the sprint test, which was conducted from a standing start on the touch pad of an electronic timing device (Newtest, Oulu, Finland). Three trials were performed, of which the fastest time was used.

#### Jump testing

Vertical jump height was measured using an Accugait force plate (Amti, USA) on the same surface for both pre- and post-testing. To reduce the influence of shoe properties on jump performance [31] players used the same shoe type (Nike, Total90 Shoot-II-IC) in all tests. A 15 min standardized warm-up procedure was used before testing, consisting of stationary biking and submaximal jumping variations. The players were instructed to stand in an upright position with feet shoulder width apart and keep hands on their hips throughout the jumps. From this position the players were instructed to do a countermovement jump by performing a rapid downward movement by flexing the knees and hips, followed by immediately extending the knees and hips in order to jump as high as possible. Jumping height was determined as the height of center of mass displacement calculated from integration (0.001 s time constant) of the vertical ground reaction force and the measured body mass [32]. Each individual test was repeated a minimum of 6–8 times until a plateau of less than 5% between two consecutive trials was reached and the best trial was then used.

#### Statistical analysis

With no predetermined level of statistical power, not-normally distributed data with distinct outliers, and a small final sample (5–9 players in each group for different outcomes), no statistical testing was performed of the dependent variables [33, 34]. Individual player data are visualized to present the distribution and non-linearity of changes. Median changes is provided as a measure of central tendency [33, 35, 36]. Means and standard deviations of group differences and combined group differences for 30 m sprint, 5- and 10 m split, and countermovement jump are presented to supply data for future sample size calculations [33, 35]. Baseline data from all randomised players that completed testing in November are displayed in Table 1.

**Table 1:** Baseline data of the intervention and control group

	Nordic group (n = 9)	Control group (n = 10)
Age (years)	23.0 ± 3.9	25.1 ± 4.9
Body mass (kg)	73.1 ± 5.8	77.9 ± 9.9
Height (m)	1.83 ± 0.05	1.81 ± 0.07
BMI (kg/m^2^)	21.8 ± 1.6	23.7 ± 2.0
Cooper test (m)	3052.5 ± 291.4	3102.5 ± 363.2
Squat 1RM load (kg)	93.6 ± 25.1	111.3 ± 8.5
30 m sprint (s)	4.101 ± 0.159	4.036 ± 0.095
5 m split (s)	0.904 ± 0.108	0.838 ± 0.027
10 m split (s)	1.686 ± 0.152	1.599 ± 0.047
CMJ (cm)	43.8 ± 3.7	42.6 ± 6.7

Group mean values and standard deviations obtained in November 2008. *BMI* body mass index, *RM* repetition maximum. The Cooper test is a 12-min running test of physical fitness. The squat was performed as a full barbell squat with the femurs parallel to the ground. Some measures involved different number of players; the Cooper test (n = 8 in both groups); the sprint test (n = 9 in the Nordic group and n = 8 in the control group); the countermovement jump test (CMJ; n = 9 in both groups); and Squat 1RM (n = 7 in the Nordic group and n = 4 in the control group)

### Results

Nineteen players were randomised and completed pre-testing (Table 1). Due to injuries (unrelated to performing the protocol), club-transfers and absence during data-collection, some players did not attend post-testing in March. Fourteen of the 19 included players completed sprint tests with all of the dropouts occurring in the control group (n = 5). Seventeen of the 19 included players completed jump test with dropouts also only occurring in the control group (n = 2). Compliance was 100%, as all players in the Nordic group performed the 27 sessions and prescribed repetitions as intended.

**Fig. 1:**
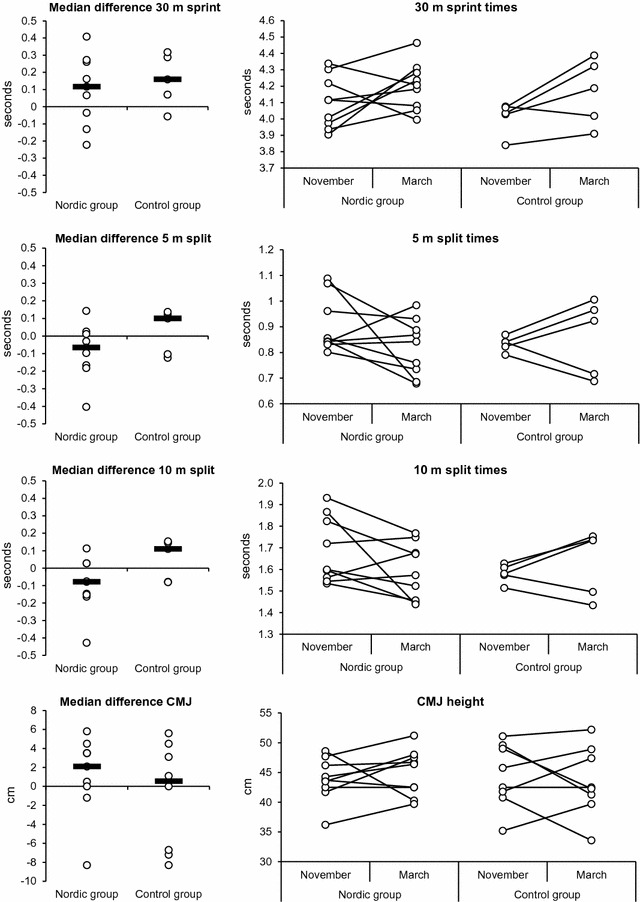
30 m sprint with 5 and 10 m split times, and Countermovement Jump height. Individual pre and post data, and median differences (black bars). *CMJ* Countermovement jump

Sprint performance on the short split distances improved for most players in the NHP (6 out of 9 improved, median changes for 5 m split: − 0.068 s; 10 m split: − 0.078 s), but not CG (2 out of 5 improved, median changes for 5 m split: + 0.1 s; 10 m split: CG: + 0.11 s), but both groups had small declines at 30 m sprint (NHP: 7 out of 9 declined, median changes: + 0.116 s; CG: 4 out of 5 declined, median changes: + 0.159 s). CMJ height mostly improved in both groups (NHP: 6 out of 9 improved, median changes: + 2.1 cm; CG: 4 out of 8 improved, median changes: + 0.55 cm) (Fig. 1).

### Discussion

Performing the full 10-week Nordic Hamstring Protocol during preseason in elite soccer players did not seem to negatively affect sprint and vertical jump performance outcomes, while in fact showing some promise for the more explosive characteristics such as the short 5 and 10 m split-times and maximal countermovement jump height compared to control group or baseline measures. The data from the present study are in line with previous findings of either maintained or increased sprint and jump performance when performing the Nordic Hamstring exercise with smaller dosage or as part of additional strength training [21–28]. Previous studies on sprint performance in elite male football players have demonstrated a difference in maximum mean 10 m sprint times between the top and bottom 25th percentile of 0.08 s [37], suggesting the median improvement of 0.078 s (mean 0.14 s) observed in the intervention group could be clinically relevant if replicated in adequately powered future trials. As such, this study can provide data for such trials to obtain appropriate statistical power and make pre-determined decisions regarding analyses (Table 2). The standard deviation of changes of, e.g. the 10 m split for both groups, were 0.13 s, so in order to show a mean between-group difference of 0.14 s, as in the present study (corresponding to a large effect size), with a power of 80% and alpha level at 0.05 using a two-tailed paired *t* test, a sample with 16 players in each group would be needed (G*Power 3.1.9.2) (Table 2).

**Table 2:** Sprint and jump performance for intervention and control group before and after mid-seasonal training period

	Nordic group			Control group			Between-group mean difference of changes
	Nov	Mar	Δ (%)	Nov	Mar	Δ (%)	
30 m (s)	4.10 ± 0.15	4.20 ± 0.14	+ 0.09 (+ 2.42%) ± 0.20	4.00 ± 0.09	4.16 ± 0.20	+ 0.15 (+3.88%) ± 0.15	0.04
5 m split (s)	0.90 ± 0.10	0.81 ± 0.10	− 0.08 (− 9.40%) ± 0.15	0.83 ± 0.02	0.85 ± 0.14	+ 0.02 (+3.21%) ± 0.12	0.10
10 m split (s)	1.68 ± 0.15	1.58 ± 0.13	− 0.09 (− 5.77%) ± 0.15	1.58 ± 0.04	1.63 ± 0.15	+ 0.05 (+3.17%) ± 0.11	0.14
CMJ (cm)	43.82 ± 3.67	44.97 ± 3.89	+ 1.15 (+ 2.63%) ± 4.20	44.47 ± 5.38	43.48 ± 5.85	− 0.98 (− 2.22%) ± 5.60	2.13

Group mean values and standard deviations. Only values for players who completed pre and post tests are presented. All between-group differences are in favor of the Nordic group. The sprint test performed was 30 meters sprint with standing start and split times after 5 and 10 meters (n = 9 in the intervention group and n = 5 in the control group). The jump test performed was a counter movement jump (CMJ; n = 9 in the intervention group and n = 8 in the control group)

The data can be of use if future research on the effect of the Nordic Hamstring exercise on either injury rates or performance outcomes in an elite football setting should come across reluctant coaching- or medical staff with reservations about impacts of the Nordic Hamstring Protocol on performance measures. The protocol of such a future trial can be designed in accordance with the present study, as it was simple to execute, and participating players and staff reported no compliance issues or adverse events, while the effort of the individual players was also deemed acceptable.

### Conclusion

Conducting the simple 10-week pre-season Nordic Hamstring Protocol in elite soccer players did not negatively affect sprint and vertical jumping performance, respectively. Signs of improved explosive acceleration characteristics as evaluated by 5 and 10 m split times, and the maximal countermovement jump height were noted, which represent highly relevant skills in top level football. The effect of Nordic Hamstring exercise on maximal acceleration, sprint and jump performance therefore should be examined more thoroughly in future large scale studies, with focus on shorter sprinting distances, and vertical jumping. Such studies can be designed based of data and other relevant information obtained during the present investigation.

## Limitations

The sample in the current study was small and warrants testing in future trials to estimate effect with adequate statistical power and room for dropouts. Only 19 players were randomised from the full team of 25 due to injuries, transfers or abstaining. Additional players dropped out during the study period for similar reasons, resulting in a high rate of dropouts from the full squad for the different outcomes (sprint: n = 11∼44%; CMJ: n = 7∼28%). It is suggested that data collection is done just prior to and after the intervention period, and preferably not spread out over several days to minimize this. As this team played in the second highest tier in Denmark and was mostly comprised of part-time professional players, conducting the study with full-time professionals might be expected to also reduce dropouts. The standard deviations of changes and individual measures were high. It does, however, seem plausible that smaller standard deviations would be observed when examining larger groups, as when looking at the distribution of individual data-points, distinct outliers can be observed. Some risk of potential bias was present, as the same author did stratification, randomisation, group allocation and managed outcome assessments, although the type of outcomes measures were objective and the author could have little to no potential impact on the assessment [38]. No clear objectives were pre-determined as currently recommended when designing pilot studies [39, 40], and no quantifiable data therefore exists on objectives such as adverse events, acceptability of effort by staff and players, muscle soreness, cost-effectiveness etc.

## Abbreviations

CONSORT: Consolidated Standards of Reporting Trials
BMI: body mass index
RM: repetition maximum
